# Expression of Concern: Abou-ElNaga et al. Novel Nano-Therapeutic Approach Actively Targets Human Ovarian Cancer Stem Cells After Xenograft into Nude Mice. *Int. J. Mol. Sci.* 2017, *18*, 813

**DOI:** 10.3390/ijms26115020

**Published:** 2025-05-23

**Authors:** International Journal of Molecular Sciences Editorial Office

**Affiliations:** MDPI, Grosspeteranlage 5, 4052 Basel, Switzerland; ijms@mdpi.com; Tel.: +41-61-683-77-34

With this notice, the IJMS Editorial Office alerts readers to concerns related to this article [1]. Following publication of a Correction [2], further concerns were raised to the Editorial Office regarding the integrity of several of the images and overall data presented in this study. The Editor-in-Chief has decided to issue this expression of concern while an investigation is being conducted. The authors of this publication have been contacted for further clarification.

Once this process has been completed, the Editorial Office will provide an update on this situation and announce any post-publication modification deemed to be necessary, as per MDPI’s Update to Published Papers policy.
